# Angiotensin-converting enzyme 2 (ACE2) mediates influenza H7N9 virus-induced acute lung injury

**DOI:** 10.1038/srep07027

**Published:** 2014-11-13

**Authors:** Penghui Yang, Hongjing Gu, Zhongpeng Zhao, Wei Wang, Bin Cao, Chengcai Lai, Xiaolan Yang, LiangYan Zhang, Yueqiang Duan, Shaogeng Zhang, Weiwen Chen, Wenbo Zhen, Maosheng Cai, Josef M. Penninger, Chengyu Jiang, Xiliang Wang

**Affiliations:** 1 State Key Laboratory of Pathogens and Biosecurity, Beijing Institute of Microbiology and Epidemiology, Beijing 100071, China; 2 Beijing 302 Hospital, Beijing, 100039, China; 3 State Key Laboratory of Medical Molecular Biology, Institute of Basic Medical Sciences, Chinese Academy of Medical Sciences, Department of Biochemistry and Molecular Biology, Peking Union Medical College, Tsinghua University, Beijing 100005, China; 4 Beijing Chao-Yang Hospital, Beijing Institute of Respiratory Medicine, Capital Medical University, Beijing 100020, China; 5 Quanzhou First Hospital, Fujian 362321, China; 6 Shishi Hospital, Fujian 362700, China; 7 Institute of Molecular Biotechnology in the Austrian Academy of Sciences, Vienna, A-1030, Austria

## Abstract

Since March 2013, the emergence of an avian-origin influenza A (H7N9) virus has raised concern in China. Although most infections resulted in respiratory illness, some severe cases resulted in acute respiratory distress syndrome (ARDS), which is a severe form of acute lung injury (ALI) that further contributes to morbidity. To date, no effective drugs that improve the clinical outcome of influenza A (H7N9) virus-infected patients have been identified. Angiotensin-converting enzyme (ACE) and ACE2 are involved in several pathologies such as cardiovascular functions, renal disease, and acute lung injury. In the current study, we report that ACE2 could mediate the severe acute lung injury induced by influenza A (H7N9) virus infection in an experimental mouse model. Moreover, ACE2 deficiency worsened the disease pathogenesis markedly, mainly by targeting the angiotensin II type 1 receptor (AT1). The current findings demonstrate that ACE2 plays a critical role in influenza A (H7N9) virus-induced acute lung injury, and suggest that might be a useful potential therapeutic target for future influenza A (H7N9) outbreaks.

Avian influenza A (H7N9) virus is a viral subtype that was detected in birds previously. However, it had not been reported in either animals or humans prior to its identification in China in March 2013^1^,^2^,^3^. The first wave of cases occurred between February and May 2013^4^,^5^,^6^. Reports of human infections then decreased during the summertime, but increased subsequently from October, demonstrating the occurrence of a second wave of infections. The disease caused by the H7N9 virus is characterized by rapidly progressing severe pneumonia. Complications include acute respiratory distress syndrome (ARDS), septic shock, and multi-organ failure that require intensive care and mechanical ventilation. To date, most influenza A (H7N9) virus-infected patient deaths were due to acute lung injury (ALI) and ARDS^7^,^8^,^9^,^10^.

The rennin-angiotensin system (RAS) is a complex network that plays a major role in maintaining blood pressure, electrolyte and fluid homeostasis, and fluid and salt balance^11^,^12^,^13^. Angiotensin-converting enzyme-2 (ACE2) was discovered as a homolog of ACE that regulated RAS negatively by converting angiotensin (Ang)-II to Ang-1–7^14^. Previous reports identified ACE2 as the receptor for the severe acute respiratory syndrome (SARS) coronavirus^15^. Recently, it was reported that ACE2 modulated innate immunity and influenced the composition of the gut microbiota^16^. Interestingly, ACE2 is also involved in the severe ALI and failure that is induced by sepsis, acid aspiration, SARS, and lethal avian influenza A H5N1 virus^17^. As such recombinant soluble ACE2 is currently being tested in phase 2 clinical trials as a potential therapy for the treatment of acute lung injury in humans^18^,^19^. Of note, we demonstrated recently that serum Ang II levels were elevated in H5N1- and H7N9-infected patients^20^. More importantly, plasma Ang II levels were linked to disease severity and predicted a fatal outcome in H7N9-infected patients^21^. Therefore, the aim of the current study was to further determine whether interfering with RAS could influence the severity of avian influenza A (H7N9) virus-induced lung injury in an experimental mouse model.

## Methods

### Animals

Four-week-old wild-type (WT) C57BL/6 (abbreviated B6) mice (Experimental Animal Center of Academy Military Medical Sciences, Beijing, China), and 4-week-old ACE2 knockout (abbreviated KO) mice (B6 background, a gift from Professor Josef M. Penninger) were housed in the animal facility at the Beijing Institute of Microbiology and Epidemiology in accordance with institutional guidelines.

All experimental protocols were approved by the Institutional Animal Care and Use Committee of Academy Military Medical Sciences (ID: SYXK2010-005). Live-virus experiments were performed in Bio-safety Level 3 facilities in accordance with governmental and institutional guidelines.

### Experimental mouse models of acute lung injury

The influenza A H7N9 virus (A/Hebei/01/2013, abbreviated Hb01/H7N9) used in this study was isolated from a confirmed H7N9-infected patient. The genomic sequences of Hb01/H7N9 are available in the Global Initiative on Sharing All Influenza Data (GISAID) database under the accession numbers EPI509120–EPI509127. Live virus experiments were performed in Biosafety Level 3 facilities in accordance with governmental and institutional guidelines. For influenza A H7N9 virus-induced acute lung injury, 4-week-old WT B6 mice were anesthetized with 50-μl 1% (w/v) pentobarbital sodium, and then inoculated intranasally (i.n.) with 2 × 10^3.5^ of the 50% tissue culture infectious dose (TCID_50_) of Hb01/H7N9 virus or mock-infected control allantoic fluid (AF). The survival, weight loss, acute pulmonary edema (wet-to-dry ratio), and histological measurements were performed as described previously^22^.

### AT1/AT2 receptor inhibitors

For inhibitor experiments, mice were injected intraperitoneally with the AT1 inhibitor losartan (15 mg/kg), the AT2 inhibitor PD123.319 (15 mg/kg), or PBS 30 min before Hb01/H7N9 virus infection.

### Angiotensin II levels and western blotting

Ang II levels were detected as described previously^21^. Rat polyclonal anti-ACE2 antibodies (R&D Systems) were used for western blotting.

### Histological examination

After being anesthetized with pentobarbital sodium, 4-week-old B6 mice were treated i.n. with 2 × 10^3.5^ TCID_50_ virus, and were then sacrificed at various days post-infection (DPI). The lungs of each group of mice (five mice per group) were fixed in formalin, and then embedded in paraffin. The number of inflammatory cells was counted, and the data are presented as the number of cell per 200× field.

### Lung wet-to-dry ratio

To assess the extent of acute pulmonary edema, the lung wet-to-dry weight ratios were calculated. WT 4-week-old B6 mice were anesthetized using sodium pentobarbital, and were then inoculated intranasally with 2 × 10^3.5^ TCID_50_ virus. At 5 DPI, the wet weights of the lungs were then measured from eight mice per group. The lungs were then heated to 68°C overnight, and the dry weights were measured.

### Quantitative real-time PCR

RNA was extracted from the lungs of 4-week-old B6 mice infected with 2 × 10^3.5^ TCID_50_ virus using an RNeasy Mini Kit (Qiagen). Real-time RT-PCR was performed using an ABI 7500 Real-Time PCR system (Applied Biosystems) with primers and a TaqMan one-step RT-PCR master mix (Applied Biosystems). Primers that could detect the influenza NP gene specifically were used. The expression of target genes was normalized to that of the control gene *GAPDH*. Relative amounts of mRNA were calculated using the comparative C_T_ method.

### Viral titration

Virus titers were measured in the supernatants of mouse lung homogenates from ACE2 KO or WT mice on day 5 post-infection. Briefly, samples were added to 96-well plates containing MDCK cells, and then diluted 10-fold. The infected cells were then cultured for 96 h. The viral titers were calculated using the Reed and Muench method, and data were expressed as log_10_ TCID_50_/g of lung tissue.

### Statistical analyses

All data are presented as means ± SEM. Measurements at single time-points were analyzed using ANOVA, and survival data were analyzed using Kaplan-Meier survival analysis. All statistical analyses were performed using the GraphPad Prism 5 software. A value of *p* < 0.05 was considered to indicate statistical significance.

## Results

### RAS is dysregulated during mouse H7N9 infection

**Figure 1:**
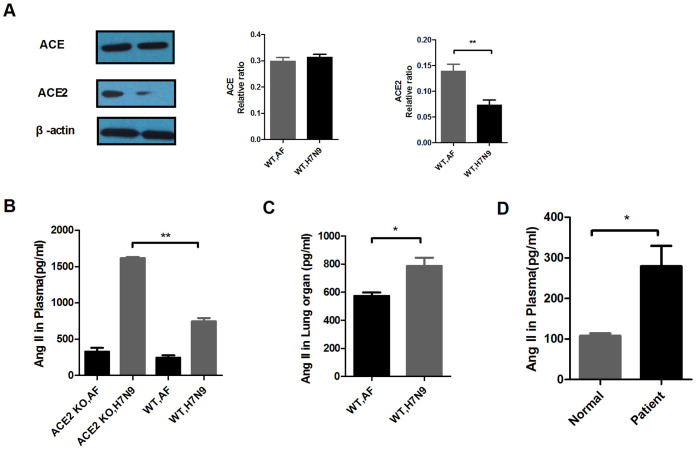
ACE2 plays a critical role in 2013 influenza H7N9 virus-induced ALI. (A) Downregulated ACE2 expression in the lungs of Hb01/H7N9 virus-infected mice. Lung tissue homogenates prepared from control and Hb01/H7N9 virus-infected WT mice on day 3 were analyzed by western blotting. (B) Plasma levels of Ang II in control and Hb01/H7N9 virus-infected ACE2 KO or WT mice on day 3 (*n* = 8). (C) Ang II levels in the lungs of control and Hb01/H7N9 virus-infected WT mice on day 3 were measured using enzyme immunoassays (*n* = 8). (D) Plasma levels of Ang II in healthy and H7N9 virus-infected patients were measured using enzyme immunoassays. *, *p* < 0.05; ** *p* < 0.01 between groups.

Two research groups cloned the ACE homolog ACE2 independently in 2000^23^. ACE2 negatively regulates RAS by inactivating Ang II^24^. To assess the effect of influenza virus infection on RAS directly, mice were infected with live influenza Hb01/H7N9 virus. Influenza A (H7N9) virus infection in WT mice caused the development of pulmonary edema and an increase in lung viral titers. Importantly, the WT mice infected with influenza H7N9 virus downregulated ACE2 protein markedly on day 3 after infection. In contrast, the expression of ACE in the lungs was comparable between H7N9-infected and control mice (Figure 1A).

Next, Ang II levels were measured in Hb01/H7N9 virus-infected mice on day 3 post-infection. Hb01/H7N9 virus infection increased Ang II levels in plasma (Figure 1B) of WT mice (*p* < 0.01) markedly. Similarly, there was also a significant increase in Ang II levels in the lungs of Hb01/H7N9 virus-infected mice (Figure 1C). It was noticeable that ACE2 protein expression was downregulated in WT mice following influenza A (H7N9) virus infection. Therefore, this experimental mouse model suggests that the ALI that was induced by influenza A (H7N9) virus infection resulted in decreased ACE2 expression and elevated Ang II levels.

To further elucidate the role of ACE2 in influenza A (H7N9) virus-induced ACI, we next examined the levels of Ang II in the plasma of H7N9-infected patients. Six patients who were PCR-positive for the 2013 influenza A (H7N9) virus were recruited from Beijing Chaoyang hospital and Quanzhou hospital, China. The characteristics underlying the conditions and the outcomes of the six patients are described in Supplementary Table S1. A significant increase in plasma Ang II levels was observed in influenza A (H7N9) virus-infected patients (Figure 1D). In addition, plasma Ang II levels were measured in influenza A (H7N9)-infected patients at different time-points post-infection, and data revealed that Ang II concentrations increased continuously increased from ~350 to 6100 pg/mL; the peak levels were detected when the patient was on the verge of death (Figure S1A). The kinetics of Ang II expression was also quantified in patients who recovered from H7N9 infection (Figure S1B; Table S2 and S3). Data revealed that plasma Ang II levels decreased sharply from the early to the late phase of influenza A (H7N9) virus infection in the recovered population. Therefore, we hypothesized that ACE2 might play a critical role in the host response against 2013 influenza A (H7N9) infection via Ang II.

### ACE2 deficiency increased the severity of H7N9-induced lung injury

**Figure 2:**
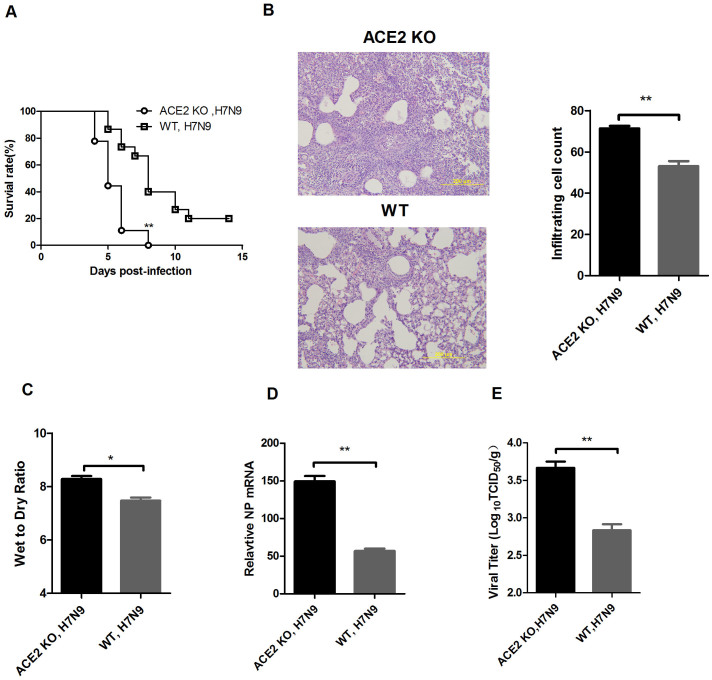
Loss of ACE2 expression worsens H7N9-induced ALI. (A) The survival rates of WT and ACE2 KO mice (*n* = 10). (B) H&E staining and infiltrating cell counts (*n* = 200 fields) in the lung tissues of WT B6 and ACE2 KO mice (*n* = 5) at day 5 post-infection. (C) The wet-to-dry ratio of lungs from WT B6 and ACE2 KO mice (n = 8) at day 5 post-infection. (D) Detection of Hb01/H7N9 virus *NP* RNA from WT B6 and ACE2 KO mice (*n* = 8) at day 5 post-infection. (E) Lung viral titers in Hb01/H7N9 virus-infected WT B6 and ACE2 KO mice (*n* = 8) at day 5 post-infection.

We next investigated whether ACE2 could mediate or protect mice from 2013 H7N9 influenza infection-induced ALI. ACE2 KO mice that were infected with influenza Hb01/H7N9 virus exhibited survival rates that were reduced significantly compared with WT mice (Figure 2A). Specifically, the survival rate of WT mice was 20%, whereas no KO mice survived for at least 9 days after influenza Hb01/H7N9 virus infection. Similarly, the lung histopathology and lung injury scores, as defined by leukocyte infiltration cell counts, were worsened significantly in ACE2 KO mice compared with WT (Figure 2B). Moreover, lung edema, defined by the wet-to-dry weight ratio of lung tissue, was much more severe in ACE2 KO mice (Figure 2C). In addition, the influenza Hb01/H7N9 virus nucleoprotein (NP) copy number was also increased greatly in the KO mice (Figure 2D). Consistent with this, the lung viral titers of the ACE KO mice were also increased significantly (Figure 2E). Therefore, these results suggest that ACE2 plays an important role in mediating H7N9 influenza-induced ALI, and that interfering with ACE2 expression might attenuate the disease severity and increase survival following respiratory H7N9 infection.

### The Ang II receptor AT1 affects the severity of H7N9-induced lung injury

**Figure 3:**
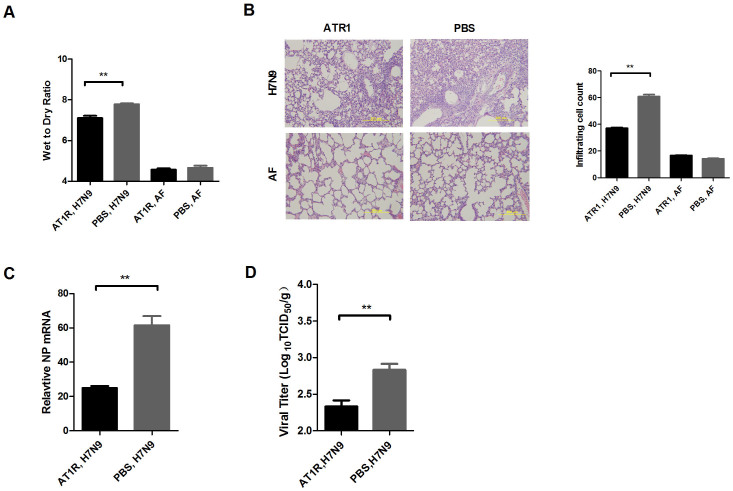
The Ang II receptor AT1 regulates H7N9-induced lung injury. (A) The wet-to-dry ratio of the lungs of WT mice treated with control or AT1 inhibitor (losartan, 15 mg/kg) 30 min before Hb01/H7N9 virus infection (*n* = 8). (B) H&E staining and infiltrating cell counts (*n* = 200 fields) in the lung tissue of Hb01/H7N9 influenza virus-infected B6 mice treated with PBS control or AT1 inhibitor (losartan, 15 mg/kg) at day 5 post-infection. (C) Detection of Hb01/H7N9 virus *NP* RNA in WT mice treated with PBS control or AT1 inhibitor (losartan, 15 mg/kg) 30 min before Hb01/H7N9 virus infection (*n* = 8) at day 5 post-infection. *NP* mRNA expression was quantified using real-time PCR, and was normalized to *GAPDH* (*n* = 10). (D) Lung viral titers in WT mice treated with PBS control or AT1 inhibitor (losartan, 15 mg/kg) before Hb01/H7N9 virus infection (*n* = 8) at day 5 post-infection. **p < 0.01(two-tailed *t*-test).

We next attempted to rescue the ALI in WT mice infected with influenza Hb01/H7N9 virus using specific AT1 and AT2 receptor blockers. Inhibiting AT1 alleviated the severity of influenza Hb01/H7N9 virus-induced lung injury significantly in WT mice, as determined by the lung wet-to-dry ratio and histopathology (Figures 3A and 3B). Similarly, the NP copy number and viral titers were decreased significantly in the lungs of H7N9 virus-infected WT mice treated with AT1 receptor blockers (Figures 3C and D). In contrast, inhibiting AT2 had no significant effect on the ALI phenotypes (Figure S2), suggesting that actions of Ang II via the AT1 receptor play a critical role in influenza A (H7N9) virus-induced ALI.

### Inhibiting AT1 attenuates H7N9-induced lung injury in ACE2 KO mice

**Figure 4:**
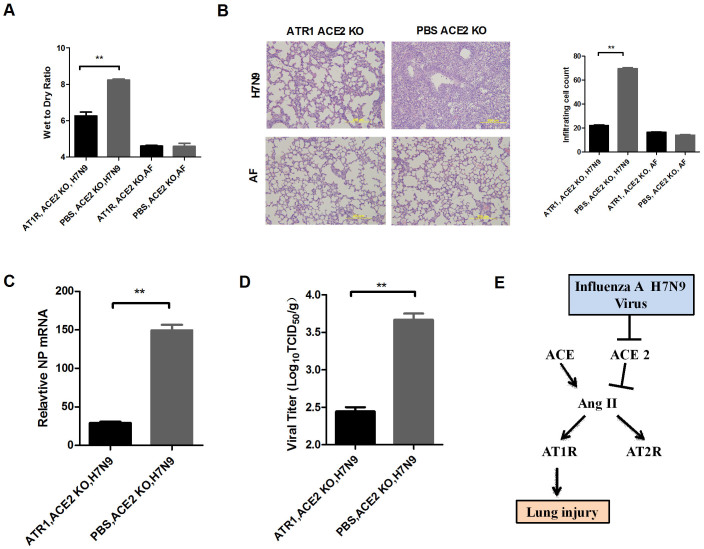
Inhibiting AT1 attenuates H7N9-induced lung injury in ACE2 KO mice. (A) The wet-to-dry ratio of the lungs of ACE2 KO mice treated with PBS control or AT1 inhibitor (losartan, 15 mg/kg) 30 min before Hb01/H7N9 virus infection (*n* = 8). (B) H&E staining and infiltrating cell counts (*n* = 200 fields) in the lung tissue of Hb01/H7N9 virus-infected ACE2 KO mice treated with PBS control or AT1 inhibitor (losartan, 15 mg/kg) at day 5 post-infection. **p < 0.01 (two-tailed *t*-test). (C) Detection of Hb01/H7N9 virus *NP* RNA in ACE2 KO mice treated with PBS control or AT1 inhibitors at day 5 post-infection. *NP* mRNA expression was assessed using real-time PCR, and was normalized to *GAPDH* (*n* = 10). (D) Lung viral titers in ACE2 KO mice treated with PBS control or AT1 inhibitor at day 5 post-infection. **p < 0.01 (two-tailed *t*-test). (E) Schematic diagram of the role of the renin-angiotensin system in ALI and influenza A H7N9 virus infection.

Similarly, the lung wet-to-dry ratio of H7N9 virus-infected ACE2 KO mice treated with At1 receptor blockers was decreased and the pathological lung alterations were improved (Figures 4A and 4B). Similarly, the NP copy number and viral titers were decreased significantly in the lungs of H7N9 virus-infected ACE2 KO mice treated with AT1 receptor blockers (Figure 4C and D). Consistent with our observations in WT mice, there was no apparent effect on lung edema and histopathology when ACE2 KO mice were treated with AT2 receptor blockers (data not shown). These results suggest that ACE2 might play a crucial role in influenza A (H7N9) virus infection-induced ALI via the AT1 receptor. Based on these data, schematic diagrams of the RAS in influenza A (H7N9) virus-induced ALI have been proposed (Figure 4E). Taken together, these data suggest that the novel influenza H7N9 virus causes severe ALI in a experimental mouse model at least in part by altering the RAS via ACE2 expression that targets AT1.

## Discussion

ARDS is the most severe stage of ALI during pathogenic infections^25^. ARDS is characterized by lung edema, increased viral titers, and the accumulation of inflammatory cells. Consistent with previous studies^21^, the current results revealed elevated levels of Ang II in plasma samples from H7N9-infected patients in China. Nevertheless, a considerably larger cohort of H7N9-infected individuals is required to confirm these observations. Influenza A (H7N9) virus-induced ALI results in the significant downregulation of ACE2, which regulates the RAS. Moreover, the current findings provide a molecular explanation for these causes of death and the mechanism of ALI in patients infected with H7N9 influenza. Ang II is upregulated after the downregulation of ACE2, and it then causes severe lung injury via the AT1 receptor during influenza A (H7N9) virus infections.

The data presented here also demonstrated that ACE2 deficiency aggravated influenza A (H7N9) virus-induced ALI in mice. Nevertheless, further studies assessing the potential therapeutic effects of recombinant human ACE2 protein are required using different animal models to verify the protective effects of ACE2 against H7N9-induced lung pathologies. The combination of clinical findings and mice experiments has revealed a critical role for the RAS in the pathogenesis of influenza A (H7N9) virus-induced ALI, and demonstrated that ACE2 plays a key role in the development and progression of H7N9 influenza. In addition, the molecular basis by which influenza H7N9 virus infection decreases ACE2 expression should be investigated in future studies. Taken together, these data raise the intriguing possibility that treatments targeting ACE2 and RAS might be viable strategies for treating influenza A (H7N9) virus infections.

## Author Contributions

P.Y., H.G., Z.Z., W.W., C.L., X.Y., L.Z., Y.D. and S.Z. contributed to experiment. P.Y., H.G. and B.C. analyzed the data. B.C., W.C., W.Z. and M.C. collected the samples. J.P., C.J. and X.W. designed the experiments and reviewed/edited the manuscript extensively. P.Y., C.J. and X.W. wrote the manuscript.

## Supplementary Material

Supplementary Information SI
