# Supplementary material for: Angiotensin-converting enzyme 2 (ACE2) mediates influenza H7N9 virus-induced acute lung injury
Source: Sci Rep. 2014 Nov 13;4:7027. doi: 10.1038/srep07027 (PMC4229671; doi:10.1038/srep07027)
Supplement: Supplementary Information — SI [file srep07027-s1.doc]

**Supplementary data to:**

**Angiotensin-converting enzyme 2 (ACE2) mediates influenza H7N9 virus-induced acute lung injury**

Penghui Yang, Hongjin Gu, Zhongpeng Zhao, Wei Wang, Bin Cao, Chengcai Lai, Xiaolan Yang, LiangYan Zhang, Yueqiang Duan, Shaogeng Zhang, Weiwen Chen, Wenbo Zhen, Maosheng Cai, Josef M. Penninger, Chengyu Jiang, Xiliang Wang

**Supplement Figures**

**
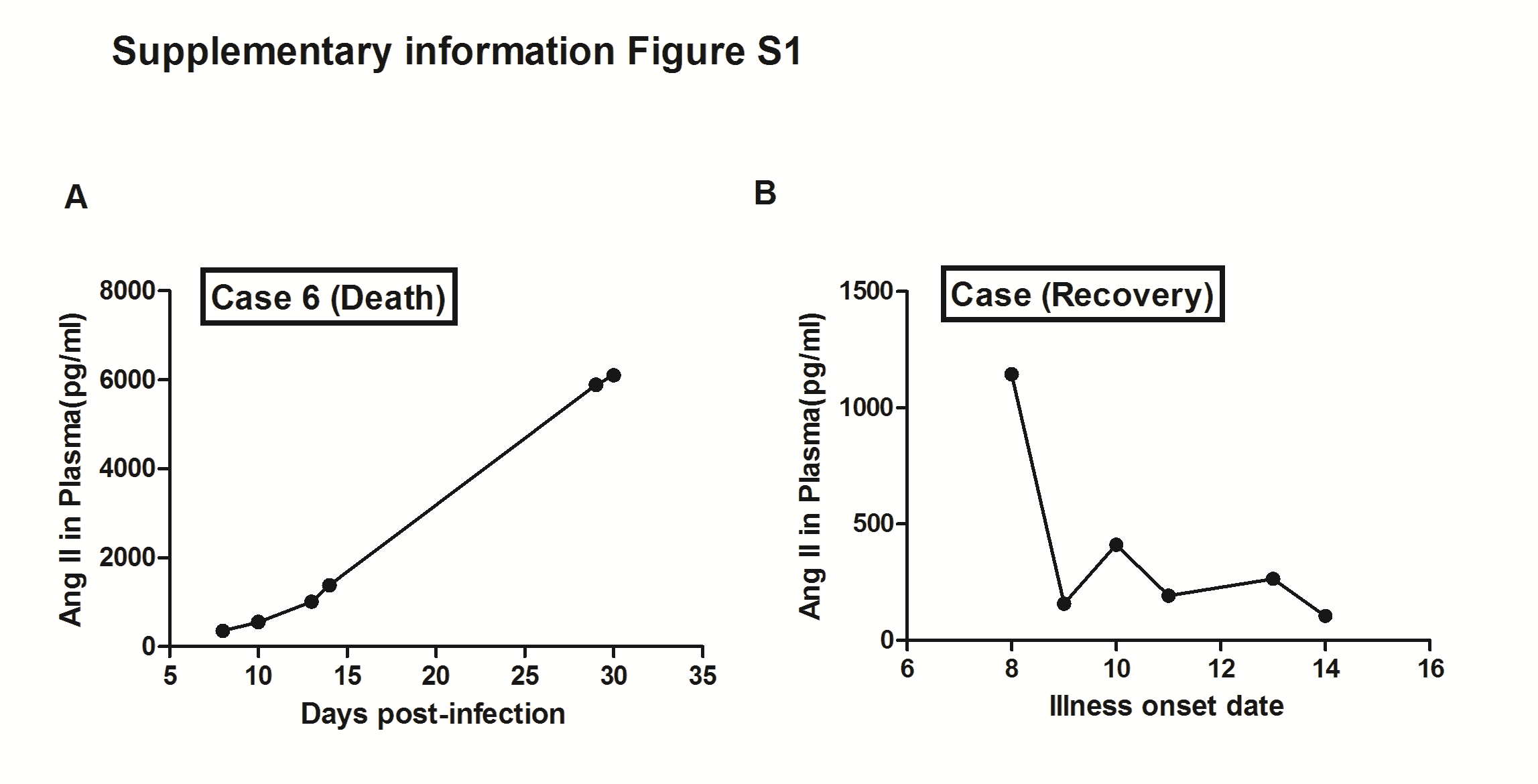
**

**Supplementary information, Figure S1** The level of plasma AngII in influenza A H7N9 confirmed infected human cases. **(A)** Angiotensin Ⅱ level in plasma from a H7N9-infected patient at different time-points post infection. **(B)** The kinetics of continued angiotensin Ⅱ level from H7N9 infected recovery patients group.

**
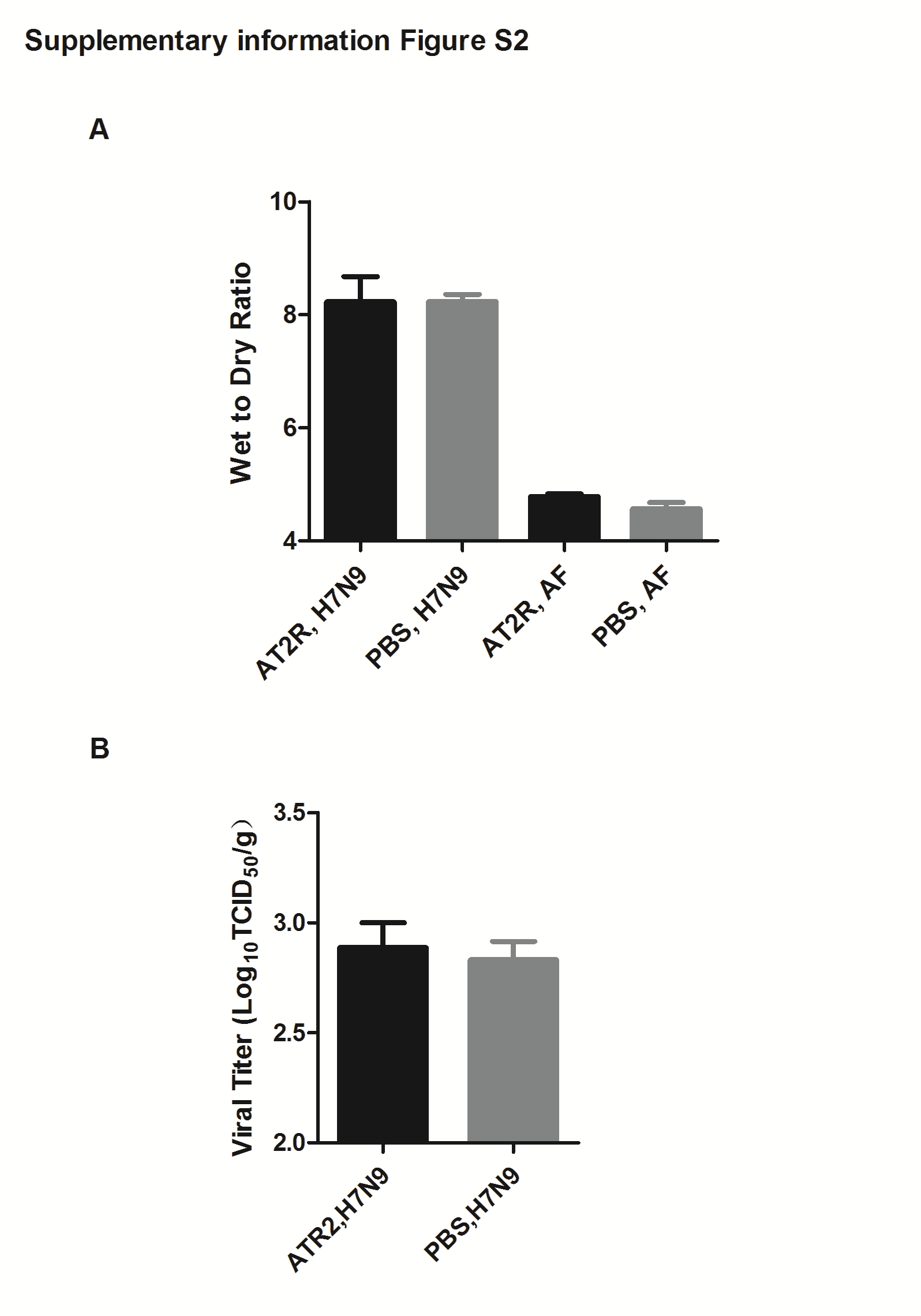
**

**Supplementary information, Figure S2** The AngII receptor AT2 do not influence H7N9 induced lung injury. (A) Wet-to-dry ratios of lungs in Hb01/H7N9 virus infected mice treated with PBS control or inhibitors to AT2 (PD123.319, 15mgkg-1) before Hb01/H7N9 virus infection (n=8). (B) Lung viral titers of Hb01/H7N9 virus infected mice treated with PBS control or inhibitors to AT2 (PD123.319, 15mgkg-1) before Hb01/H7N9 virus infection (n=8).

**Supplementary Tables**

**Supplementary Table 1 Detailed information of 6 influenza A H7N9 infected patients recruited**

| Detailed information of 6 infected patients recruited | | | | | | |
| --- | --- | --- | --- | --- | --- | --- |
| Samples | Age | Gendar | Blood-collecting date | Illness Onset date | AngⅡ Conc（pg/ml) | Outcome |
| Case 1 | 60 | Male | 2014-01-23 | 13 | 264.9 | recovery |
| Case 2 | 50 | Male | 2014-01-23 | 8 | 1143.8 | recovery |
| Case 3 | 55 | Male | 2014-01-27 | 11 | 191.4 | recovery |
| Case 4 | 30 | Male | 2014-01-26 | 13 | 368.8 | death |
| Case 5 | 36 | Male | 2014-02-10 | 10 | 410.6 | recovery |
| Case 6 | 61 | Female | 2013-07-19 | 8 | 356.8 | death |

**Supplementary Table 2 Angiotensin Ⅱ level from A H7N9 infected patients at different time-points**

| Blood-collecting date | Illness Onset date | AngⅡ Conc（pg/ml) |
| --- | --- | --- |
| 2013-07-19 | 8 | 356.8 |
| 2013-07-21 | 10 | 556.1 |
| 2013-07-24 | 13 | 1010.6 |
| 2013-07-25 | 14 | 1380.6 |
| 2013-08-09 | 29 | 5891.4 |
| 2013-08-10 | 30 | 6104.8 |

**Supplementary Table 3 Kinetics of continued angiotensin Ⅱ level from H7N9 infected recovery patients**

| Samples | Blood-collecting date | Illness Onset date | AngⅡ Conc（pg/ml) |
| --- | --- | --- | --- |
| Case 2 | 2014-01-23 | 8 | 1143.8 |
|  | 2014-01-24 | 9 | 156.3 |
| Case 5 | 2014-02-10 | 10 | 410.6 |
| Case 3 | 2014-01-27 | 11 | 191.4 |
| Case 1 | 2014-01-23 | 13 | 264.9 |
|  | 2014-01-24 | 14 | 104.8 |
